# Vaccination prevents hospitalisations of children and adolescents. A real-world analysis

**DOI:** 10.1016/j.lana.2022.100351

**Published:** 2022-08-17

**Authors:** Pablo G. Bolcatto

**Affiliations:** a Instituto de Matemática Aplicada del Litoral (IMAL, CONICET/UNL), FHUC. Santa Fe 3000, Argentina; b Instituto de Investigaciones Científicas y Técnicas para la Defensa (CITEDEF), 1603 Buenos Aires, Argentina

Facing a new disease is the biggest challenge for people, governments, and societies in general on a planetary scale in this century. The necessity and urgency to understanding its evolution, effects, and consequences is a global imperative. Clinicians and health policy deciders have to work in parallel with the profuse emerging scientific evidence. In particular, it is remarkable the way in which many laboratories and agencies put on efforts to reach effective vaccines in very short times. Thus, governments must design vaccination strategies in order to reach massive immunity.^1^

In this issue of The Lancet Regional Health – Americas, S. González, S. Olszevicki et al.^2^ give light regarding the results of a public policy carried out in a particular state of Argentina (Province of Buenos Aires) on two groups: children in the range of 3-11 years old and adolescents of 12-17 years old.

Argentina's government decided to vaccinate adolescents with two vaccines with modified RNA technologies: mRNA-1273 (Spikevax) from Moderna and BNT162b2 (Comirnaty) from Pfizer-BioNTech.^3^ The campaign started in August 2021. Besides, in October 2021 the paediatric subpopulation began to be inoculated with BBIBP-CorV (Sinopharm), an inactivated virus technology vaccine.^3^ The cohort for both groups include patients who had two doses by 1 December 2021 with a monitoring window from 14 December 2021 to 9 March 2022. This period can be split: the first with the coexistence of Delta and Omicron variants of concern (VOC) and a later one in which only the Omicron variant is prevalent.

While laboratory studies reported that RNA-based vaccines have a major percentage of effectiveness than those of inactivated virus (Refs. 19-27 in Ref. [2]), the question for policymakers is what to do during a global emergency situation. This is particularly critical for the paediatric subpopulation. Health policy decisions include non-pharmacological strategies which could impact other issues of the health and growth of young people.^1^

Within such context, the observational real-world analysis reported in Ref. [2] allows to extract very interesting conclusions. Firstly, attending to the impossibility to do long-term analysis or controlled laboratory experiments they choose the Vaccination Effectiveness (VE) as a signature of the protection of the vaccination scheme against hospitalisations for the 3-17 age population. As in Argentina, the health system covered the requirements of attention in all the COVID19 pandemic, the number of children and adolescents hospitalised become a hard data for this purpose.

As overall result, effectiveness (adjusted VE value) for the 3-11 age (BBIBP-CorV) subgroup was 76·4% [62·9-84·5] and 80·0% [64·3-88·0] for the 12-17 age (mRNA-1273 and BNT162b2 vaccines) subgroup. These results are in line with previous analyses (Refs. 22-27 in Ref [2]). Moreover, vaccination was effective against hospitalisations for all the subgroups analysed. However, there is a clear difference between the period when Delta and Omicron variant coexist (VE = 82.7%) and the Omicron outbreak (VE = 67.7%).

As should occur in good scientific work, beyond the questions solved, the emergence of new open questions is desirable and necessary. The paper by S. González, S. Olszevicki et al.^2^ fits this requirement. For example, quite surprisingly there is a clear difference in VE when gender is compared. Females have major protection against hospitalisations (VE = 84.1%) in comparison with the Male subgroup (VE = 69.6%), being this difference more pronounced than in other subgroups analysed (such as age and comorbidities).

Another point is the noticeable difference in VE for the two subperiods of the time interval used for the analysis: Why is the VE more significant when Delta and Omicron variants coexist than when only Omicron is prevalent? Is this due to the minor effectiveness of the vaccine against the Omicron variant, or does it refer to a short-time immunity yielding global waning protection to the population? Perhaps the answer is a combination of both, but it is quite clear that a continuous redesigning of the vaccination campaigns becomes necessary to the policy deciders. In this sense, the authors suggest that a booster dose in the subpopulation considered can be a good strategy to ensure a persistent immunity to the whole population. Real-world studies, laboratory experiments, theoretical model supports [^4^ and references therein] are surely proper tools for this purpose.

In conclusion, the work of S. Gonzalez, S. Olszevicki et al.^2^ gives valuable information to understand the effects of vaccination in children and adolescents. Vaccination is effective against hospitalisations for all subgroups despite the new prevalent variants or the technology of the vaccine. Nowadays, many countries report increasing evidence about the circulation of new subvariants or lineage under monitoring (VOC-LUM) such as BA.2.12.1, BA.4, and BA.5.^5^ Therefore, new vaccination strategies should be implemented to improve the effectiveness against the dominant VOC regardless of the new VOC-LUM. In this sense, Argentina's health minister has recently authorised a booster dose of mRNA-1273 in children, and even starts a vaccination scheme for the 6-months to 3-years-old subgroup.

## Declaration of interests

I declare do not have any conflict of interest with the authors or institutions involved in the commented paper.
